# Evaluation of a radioimmunoassay for neuron specific enolase in small cell lung cancer.

**DOI:** 10.1038/bjc.1985.198

**Published:** 1985-09

**Authors:** E. H. Cooper, T. A. Splinter, D. A. Brown, M. F. Muers, M. D. Peake, S. L. Pearson

## Abstract

A radioimmunoassay for neuron specific enolase (NSE), a marker of neuroendocrine differentiation, has been evaluated in small cell lung cancer (SCLC). In untreated patients 25/38 (68%) with localized SCLC had raised blood levels of NSE (greater than 13 ng ml-1), in extensive disease 34/39 (87%) patients had raised NSE levels. In patients with non-small cell lung cancer (NSCLC) the serum levels were raised in 16/94 (17%). In extensive tumours of non-pulmonary origin NSE levels were increased in 24/116 (20%) patients. Longitudinal studies indicated a good correlation between the response to chemotherapy and fall of NSE levels. Tumour progression was accompanied by a rising NSE in 25/29 patients, with doubling times of 7-90 days. In patients with progression with a normal NSE the recurrence was a NSCLC. Cerebral metastases occurring as the only recurrence during clinical complete remission were not accompanied by a rise of NSE. Serum NSE levels provides a valuable monitor for SCLC during and after chemotherapy.


Br. J. Cancer (1985), **52**, 333–338

### Evaluation of a radioimminoassay for neuron specific enolase in small cell lung cancer

E.H. Cooper[1], T.A.W. Splinter[2], D.A. Brown[1], M.F. Muers[3], M.D. Peake[4] & S.L. Pearson[3]

[1]*Unit for Cancer Research, University of Leeds, Leeds LS2 9JT, UK;* [2]*University Hospital, Dijkzigt, Rotterdam 3015GD, The Netherlands;* [3]*Killingbeck Hospital, Leeds; and* [4]*Pontefract Infirmary, Yorkshire, UK.*

**Summary** A radioimmunoassay for neuron specific enolase (NSE), a marker of neuroendocrine differentiation, has been evaluated in small cell lung cancer (SCLC). In untreated patients 25/38 (68%) with localized SCLC had raised blood levels of NSE (>13 ng ml$^{-1}$), in extensive disease 34/39 (87%) patients had raised NSE levels. In patients with non-small cell lung cancer (NSCLC) the serum levels were raised in 16/94 (17%). In extensive tumours of non-pulmonary origin NSE levels were increased in 24/116 (20%) patients. Longitudinal studies indicated a good correlation between the response to chemotherapy and fall of NSE levels. Tumour progression was accompanied by a rising NSE in 25/29 patients, with doubling times of 7–90 days. In patients with progression with a normal NSE the recurrence was a NSCLC. Cerebral metastases occurring as the only recurrence during clinical complete remission were not accompanied by a rise of NSE. Serum NSE levels provides a valuable monitor for SCLC during and after chemotherapy.

Enolase is a glycolytic enzyme [EC 4.2.1.11] that is composed of sub units α, β & γ. Neuron specific enolase [NSE] is the γγ dimer of enolase (Marangos *et al.*, 1979). There is growing evidence that the histochemical demonstration of immuno-reactive neuron specific enolase (NSE) in tumour cells can be used as an indicator of their likely neural or neuroendocrine origin. Biochemically these cells are characterised as possessing amine precursor uptake and decarboxylation (APUD) enzyme systems and the production of various hormones and peptides.

Tumours arising from the neuroendocrine cell system that contain NSE include, small cell lung cancer (SCLC) (Marangos *et al.*, 1982; Sheppard *et al.*, 1984; Springhall *et al.*, 1984) and some comparative rare cancers such as neuroblastoma (Odelstad *et al.*, 1982; Tsokos *et al.*, 1984; Zeltzer *et al.*, 1983) pancreatic islet cell cancers (Simpson *et al.*, 1984; Lloyd *et al.*, 1984) carcinoid tumours (Sheppard *et al.*, 1984) and medullary thyroid carcinoma (Lloyd *et al.*, 1983). Several immuno-assay methods have been described for NSE in body fluids. The results of assays of serum NSE using the radioimmunoassay (RIA) devised by Marangos and his colleagues have been published for several types of tumours of neuroendocrine origin (Carney *et al.*, 1982; Prinz *et al.*, 1983; Zeltzer *et al.*, 1983). Other assays include further RIAs (Pahlman *et al.*, 1984; Royds *et al.*, 1984) including assays depending on the cross reactivity of an anti-rat NSE antiserum and human NSE (Kato *et al.*, 1983), or anti-mouse NSE and human NSE (Akoun *et al.*, 1985) and immunobioluminescence assays (Wevers *et al.*, 1983; Gerbitz *et al.*, 1984).

Small cell lung cancer is the commonest type of tumour showing neuroendocrine differentiation. The measurement of serum NSE has shown to be of value in monitoring SCLC (Carney *et al.*, 1982; Pahlman *et al.*, 1984; Esscher *et al.*, 1985; Kato *et al.*, 1983; Akoun *et al.*, 1985; Ariyoshi *et al.*, 1983; Johnson *et al.*, 1984).

Recently Pharmacia, Sweden have introduced a radioimmunoassay which provides the opportunity for a wide based large scale assessment of the role of NSE measurements in oncology. This paper describes an evaluation of the Pharmacia NSE-RIA assay in the assessment and monitoring of SCLC.

### Materials and methods

*Patients* One hundred and seventy one patients with lung cancer were examined at presentation before treatment. They included 77 SCLC and 94 non-small cell carcinoma of the lung (NSCLC) (11 adeno- 69 squamous, and 15 large cell). Patients were staged as having limited disease (disease confined to one hemithorax and mediastinal lymph-nodes) or extensive disease (any spread outside these regions).

Various control groups were examined including 33 blood donors, 20 advanced cancers of breast and gastrointestinal origin with liver metastases, 42 disseminated prostatic cancer, 45 benign gastro-

Correspondence: E.H. Cooper
Received 15 April 1985; in revised form 18 May 1985.

© The Macmillan Press Ltd., 1985

intestinal disease, 20 bronchopneumonia, 14 patients with benign lesions of the lung that were considered as possible lung cancers prior to biopsy and 10 patients with lung metastases from distant sites.

Longitudinal studies were made of specimens obtained from 37 patients with SCLC, 19 from the start of treatment, and 18 commenced at various times after initiating chemotherapy. The follow up period was 4–28 months (average 10.5 months) with the sampling generally every 4–6 weeks according to the treatment protocol. The total samples per patient varied from 6 to 19. Thirty three patients were treated with CAVP (Cyclophosphamide 1000 mg m$^{-2}$ day 1 i.v. Adriamycin 45 mg m$^{-2}$ day 1 i.v., VP 16:213 100 mg m$^{-2}$ days 1, 3 and 5 i.v.) repeated every 3 weeks. In these studies commenced in 1982, samples of heparin plasma had been stored at each visit to the clinic. A further four patients were studied longitudinally, three were treated with ifosfamide, VP 16:213 and vincristine, and one received radiotherapy alone; in these patients serum was measured.

The serum and heparin plasma samples were stored at −20°C prior to analysis. Haemolysed samples were not used as they could be a source of a false elevation of NSE (Pahlman *et al.*, 1984, Johnson *et al.*, 1984).

An estimate of the inflammatory response to the combined effects of tumour tissue destruction and infection was obtained from the serum C-reactive protein (CRP) level measured by radial immunodiffusion.

The Pharmacia NSE RIA test is a double antibody radio-immunoassay. NSE in the sample competes with a fixed amount of $^{125}$I-labelled NSE for the binding sites of the specific antibodies (present in limited amount). Bound and free NSE are separated by the use of a second antibody covalently bound to spherical particles of agarose. After addition of the agarose antibody derivative, the mixture is centrifuged. The supernatant with the free NSE is then separated from the agarose pellet containing the bound NSE by decanting. The removal of the liquid phase can be done quantitatively and without extra washing. The radioactivity in the pellet is then measured. It is inversely proportional to the quantity of NSE in the sample. The detection limit is <2.6 ng ml$^{-1}$, the measuring range 3.2–260 ng ml$^{-1}$ with an average analysis time of ~6 h for a batch of 40 samples. The suggested upper limit of normal is 12.5 ng ml$^{-1}$.

### Results

The mean ±s.d. serum NSE level in 33 blood donors was 9.3±2.1 ng ml$^{-1}$. The NSE–RIA test showed a coefficient of variation of 12.5% between assays and 9.8% within assays over a range of 4–100 ng ml$^{-1}$. The manufacturer stated that the kit has a coefficient of variation of 7.6% between assays and 5.8% within assay for a serum with an NSE content of 13.6 ng ml$^{-1}$.

*Presentation data*

The distribution of NSE levels in patients with SCLC and NSCLC, subdivided according to histological type and extent of the disease are shown in Figure 1. These are contrasted to the NSE levels in patients with lung lesions considered as possible lung cancer and demonstrated to be benign. It is evident that the levels of NSE in SCLC are generally considerably higher than in other forms of lung cancer. The median serum NSE concentration for SCLC was 14 ng ml$^{-1}$ and 42 ng ml$^{-1}$ in patients with limited and extensive disease respectively. This contrasts to a median level of 7.2 ng ml$^{-1}$ and 9.1 ng ml$^{-1}$ in patients with limited and extensive NSCLC respectively. The incidence of raised levels of NSE>13 ng ml$^{-1}$ in other forms cancer and benign disease was low as shown in Table I. In bronchopneumonia and all forms of cancer there was no correlation between the CRP level and the serum NSE level ($r$=0.04, $P$=0.78). The median level was 7.5 ng ml$^{-1}$ in 11 patients with lesions suspicious of lung cancer that were

**Figure 1** Distribution of NSE levels in patients with lung cancer at presentation. (●) serum; (○) heparin plasma. L=localized disease; E=extensive disease.

**Table I** NSE levels in lung cancer, various benign and malignant diseases and blood donors

| *Serum NSE ng ml*$^{-1}$ | *<13* (%) | *13–25* (%) | *25–50* (%) | *>50* (%) |
|---|---|---|---|---|
| Blood donors (33) | 31 (94) | 2 (6) | | |
| SCLC pre-treatment | | | | |
| limited disease (38) | 13 (34.3) | 9 (23.7) | 11 (28.9) | 5 (13.2) |
| extensive disease (39) | 5 (12.8) | 4 (10.2) | 12 (30.8) | 18 (46.2) |
| Non SCLC (94) | 78 (83) | 9 (9.5) | 3 (3.2) | 4 (4.2) |
| Metastatic tumours in the lung (10) | 8 (80) | 2 (20) | | |
| Benign lesions of lung (11) | 10 (91) | 1 (9) | | |
| Bronchopneumonia (20) | 13 (65) | 6 (30) | 1 (5) | |
| Benign gastrointestinal and liver disease (45) | 38 (84.4) | 7 (15.6) | | |
| Breast and gastrointestinal cancer with hepatic mets. (20) | 17 (85) | 3 (15) | | |
| Metastatic carcinoma of prostate (42) | 31 (73.8) | 8 (19) | 3 (7.2) | |

eventually shown to be benign and 9.4 ng ml$^{-1}$ in bronchopneumonia. In 10 patients with lung metastases, from a variety of primary tumours, the median level was 5.6 ng ml$^{-1}$, and all were <15 ng ml$^{-1}$.

*Longitudinal studies*

Several patterns of response could be recognised, these were identified in the 23 patients who were studied from presentation the rarest being persistently high NSE after giving chemotherapy; 2 out of 23 (8.6%) of cases observed from presentation demonstrated this pattern. More commonly the NSE level gradually fell to reach a plateau after 2–6 courses of chemotherapy. In 4 patients the level fell to normal after a single course of chemotherapy. Several patients showed oscillating levels initially during the first few courses of treatment, NSE levels subsequently reached a low plateau. An example of the rapid fall patterns is shown in Figure 2, the gradual decline of NSE during chemotherapy is shown in Figure 3. The patient treated by radiotherapy alone had an NSE that fell from 40 ng ml$^{-1}$ to 7.0 ng ml$^{-1}$ within 1 month of treatment.

Nine patients including the 4 who responded rapidly were judged as being in complete remission after ending their chemotherapy, they were then followed for 6–22 months. Six out of 68 NSE (8.8%) measurements in these patients were unexpectedly high on a single occasion but the subsequent observations were all <15 ng ml$^{-1}$ whilst the patients remained in clinical complete remission.

In 8 patients who developed brain metastases at a time when the disease had been in clinical remission the NSE levels remained normal.

Twenty-five out of 29 patients (15 of whom had been followed from presentation) who were

**Figure 2** Serial NSE levels in a patient with extensive SCLC. After one course of chemotherapy NSE levels rapidly falls to normal levels, reaches a plateau for 90 days and then rises with a doubling time of 34 days. No response is induced by the original chemotherapy 6 or salvage chemotherapy 7.

clinically progressive with disease outside the brain had exponentially rising NSE levels, which preceded the clinical detection of progressive disease by 0–112 days (median 42 days). Examples of the rates of change of NSE are shown in Figure 4 the doubling time of those illustrated were 20–50 days, but the extremes were 7–90 days. Three of these 29 patients had a relapse confirmed histologically to be of a NSCLC, and one patient had clinical

**Figure 3** Serial NSE levels in a patient with limited SCLC. After the start of chemotherapy NSE first rises, then slowly declines. After 4 courses of chemotherapy the NSE reaches a plateau for 197 days and then rises with a doubling time of 88 days. Radiotherapy given to the tumour and prophylatically to the brain.

**Figure 4** A comparison of the rates of increase of NSE during the evolution of relapsed SCLC.

progressive disease without a concomitant rise of NSE.

The serial measurement of CRP did not provide a sensitive tumour marker, but confirmed that the NSE levels were unaffected by infection.

### Discussion

A raised serum NSE or heparin plasma ($>13\,ng\,ml^{-1}$) as assayed by the Pharmacia NSE-RIA was observed in 77% of patients with SCLC at presentation. A comparison of the present data with published series is shown in Table II. Showing that the test gives results similar to those reported using a variety of immunoassays for NSE. Large cell undifferentiated lung cancers, which may be confused with SCLC are shown to have high NSE levels in this study and by Ariyoshi et al. (1983) and Pahlman et al. (1983). Therefore a higher cut off value than in the present study would seem advisable when using the test to indicate that a tumour may be a SCLC in those cases where the biopsy was equivocal, technically unsatisfactory or

**Table II** Frequency of raised serum NSE levels in SCLC and NSCLC at presentation

| Author ref. | Upper limit of normal (*ng ml*$^{-1}$) | *SCLC* Limited (%) | Extensive | *NSCLC* (%) |
|---|---|---|---|---|
| Carney *et al.* (1982) | 12 | 15/38 (39) | 49/56 (87) | — |
| Johnson *et al.* (1984) | 20 | 23/39 (59) | 45/54 (83) | — |
| Esscher *et al.* (1985) | 12 | 34/48 (71.8) | 54/55 (98.2) | 13/51 (25.4) |
| Ariyoshi *et al.* (1983, 1984) | 10 | 6/13 (46) | 24/27 (88.8) | 21/125 (16.8) |
| Akoun *et al.* (1985) | 10 | 6/16 (37.5) | 22/27 (78.6) | 2/14 (14.2) |
| This study | 13.5 | 25/38 (68.5) | 34/39 (68.5) | 16/94 (17) |

unobtainable. In such a case NSE levels $>25\,\text{ng ml}^{-1}$, are highly suspicous but not an infallible diagnostic indicator of SCLC.

Treatment of SCLC by chemotherapy was followed by a decrease of NSE when it had been raised at presentation to reach normal levels when there was clinical evidence of an objective remission. By contrast the levels in patients who only had stable disease or failed to respond to treatment did not normalise. The NSE level was unaffected by the presence of cerebral metastases if they were the solitary site of progression of the disease. All patients except four who had progressive disease had an exponential rise of NSE, which could even be detected as it commenced its rise through the normal range. Retrospectively, the interval between the first rise of NSE and the clinical detection of progression varied from 0–112 days (median 42, $n=14$). This data supports the report of Johnson *et al.* (1984) who showed a persistently raised NSE level in 15 out of 23 (65%) patients 2–12 weeks (median 4) before clinical recognition of recurrence. It is of interest that 3 of the patients, who showed progressive disease without a rise of NSE, had histologically proven NSCLC. This indicates that normal NSE levels in a case of progressive disease warrant further investigation of the nature of the recurrent tumour. The relationship between tumour mass and NSE and response to treatment seen in the present study is comparable to that reported by Carney *et al.* (1982); Ariyoshi *et al.* (1983; 1984); Pahlman *et al.* (1984); Johnson *et al.* (1984) and Akoun *et al.* (1985).

Immunochemical assays for NSE either use an antisera to human $\gamma\gamma$ enolase (NSE) or depend on the cross reactivity of anti rat $\gamma\gamma$ enolase (Kato *et al.*, 1983) or anti mouse $\gamma\gamma$ enolase (Akoun *et al.*, 1985) antisera with human NSE. However, it is evident that the serum contains a mixture of the $\gamma\gamma$ form of enolase and $\alpha\gamma$ hybrid molecules that cross react with the antisera to $\gamma\gamma$ enolase. Gerbitz *et al.* (1984) have estimated that the serum concentrations in healthy subjects of the $\alpha\gamma$ and $\gamma\gamma$ enolase were 6.0 $\pm 3.2$ and $3.3\pm1.5\,\text{ng ml}^{-1}$ respectively. Kato *et al.* (1983) reported the concentrations to be $4.1\pm1.4$ and $1.5\pm0.4\,\text{ng ml}^{-1}$. In some patients with SCLC Gerbitz *et al.* (1984) reported increases of $\gamma\gamma$ enolase from 2 to 20 fold; minor changes occurred in the $\alpha\gamma$ enolase level in 6 out of the 7 patients but in 1 of them the $\alpha\gamma$ form was considerably increased. There is still uncertainty whether the cross-reactivity is important in the interpretation of the NSE test. The present data and published information suggests that the current tests perform well as indicators of large tumour burdens.

This study of the Pharmica NSE-RIA test has again confirmed the potential of NSE as a marker for the monitoring of SCLC during and especially after treatment. Further prospective studies will be needed to determine whether it should be taken into account when deciding the optimal number of cycles of chemotherapy that are required, or can be a reliable and early indicator of recurrence once chemotherapy has been stopped. The introduction of commercial NSE assays will help in the build up of a substantial experience of NSE measurements in clinical oncology that are needed at this time. No doubt in lung cancer such an investigation should not only include SCLC but other forms where the histological differentiation is equivocal.

We are grateful to Pharmacia Health Care AB, Uppsala, Sweden for supplying the NSE-RIA kits for evaluation and to Dr K. Hiesche for his advice and Mrs C. Batten for her secretarial assistance.

### References

AKOUN, G.M., SCARNA, H.M., MILLERON, B.J., BENICHOU, M.P. & HERMAN, D.P. (1985). Serum neuron-specific enolase. A marker for disease extent and response to therapy for small-lung cancer. *Chest*, **87**, 39.

ARIYOSHI, Y., KATO, K., ISHIGURO, Y., OTA, K., SATO, T. & SUCHI, T. (1983). Evaluation of serum neuron-specific enolase as a tumor marker for carcinoma of the lung. *Gann.*, **74**, 219.

ARIYOSHI, Y. (1984). Evaluation of neuron-specific enolase as a new tumour marker for carcinoma of the lung. *Gan. No. Rinsho.*, **30**, 569.

CARNEY, D.N., IHDE, D.C., COHEN, M.H. & 4 others. (1982). Serum neuron-specific enolase: A marker for disease extent and response to therapy of small-cell lung cancer. *Lancet*, **i**, 583.

ESSCHER, T., STEINHOLTZ, BERGH, J., NOU, E., NILSSON, K. & PAHLMAN, S. (1985). Neurone specific enolase: A useful dignostic serum marker for small cell carcinoma of the lung. *Thorax*, **40**, 85.

GERBITZ, K-D., SUMMER, J. & THALLMER, J. (1984). Brain-specific proteins: Solid-phase immunobioluminescence assay for neuron-specific enolase in human plasma. *Clin. Chem.*, **30**, 382.

JOHNSON, D.H., MARANGOS, P.J., FORBES, J.T. & 4 others (1984). Potential utility of serum neuron-specific enolase levels in small cell carcinoma of the lung. *Cancer Res.*, **44**, 5409.

KATO, K., ASAI, R., SHIMIZU, A., SUZUKI, F. & ARIYOSHI, Y. (1983). Immunoassay of three enolase isozymes in human serum and in blood cells. *Clinica. Chemica. Acta.*, **127**, 353.

LLOYD, R.V., SISSON, J.C. & MARANGOS, P.J. (1983). Calcitonin, carcinoembryonic antigen and neuron-specific enolase in medullary thyroid carcinoma. *Cancer*, **51**, 2234.

LLOYD, R.V., MERVAK, T., SCHIMDT, K., WARNER, T.F.C.S. & WILSON, B.S. (1984). Immunohisto-chemical detection of chromogranin and neuron-specific enolase in pancreatic endocrine neoplasms. *Am. J. Surg. Pathol.*, **8**, 607.

MARANGOS, P.J., SCHMECHEL, D. & PARAMA, A.M. (1979). Measurement of neuron-specific (NSE) and non-neuronal (NNE) isoenzymes of enolase in rat, monkey and human nervous tissue. *J. Neurochem.*, **33**, 319.

MARANGOS, P.J., GAZDAR, A.F. & CARNEY, D.N. (1982). Neuron specific enolase in human small cell carcinoma cultures. *Cancer Lett.*, **15**, 67.

ODELSTAD, L., PAHLMAN, S., LACKGREN, G., LARSSON, E., GROTTE, G. & NILSSON, K. (1982). Neuron specific enolase: A marker for differential diagnosis of neuroblastoma and Wilms' tumor. *J. Ped. Surg.*, **17**, 381.

PAHLMAN, S., ESSCHER, T., BERGH, J., STLINHOLTZ, L., NOU, E. & NILLSON, K. (1984). Neuron specific enolase as a marker for neuroblastoma and small cell carcinoma of the lung. *Tumor Biol.*, **5**, 119.

PRINZ, R.A., BERMES, E.W., KIMMEL, J.R. & MARANGOS, P.J. (1983). Serum markers for pancreatic islet cell and intestinal carcinoid tumors: A comparison of neuron-specific enolase β-human chorionic gonadotropin and pancreatic polypeptide. *Surgery*, **94**, 1019.

ROYDS, J.A., LILLEYMAN, J.S., TIMPERELEY, W.R. & TAYLOR, C.B. (1984). Cerebrospinal fluid enolase isoenzymes and neurotoxicity in early treatment of lymphoblastic leukaemia. *Arch. Dis. Child.*, **59**, 266.

SHEPPARD, M.N., CORRIN, B., BENNET, M.H., MARANGOS, P.J., BLOOM, S.R. & POLAK, J.M. (1984). Immunohistochemical localisation of neuron specific enolase (NSE) in small cell carcinomas and carcinoid tumours of the lung. *Histopathology*, **8**, 171.

SIMPSON, S., VINK, A.I., MARANGOS, P.J. & LLOYD, R.V. (1984). Immunohistochemical localization of neuron-specific enolase in gastroenteropancreatic neuroendocrine tumors. *Cancer*, **54**, 1365.

SPRINGALL, D.R., LACKIE, P., LEVENE, M.M., MARANGOS, P.J. & POLAK, J.M. (1984). Immunostaining of neuron-specific enolase is a valuable aid to the cytological diagnosis of neuroendocrine tumours of the lung. *Pathology*, **143**, 259.

THOMPSON, R.J. & DAY, I.M.N. (1982). Measuring serum neuron-specific enolase. *Lancet*, **i**, 1126.

TSOKOS, M., LINNOILA, R.I., CHANDRA, R.S. & TRICHE, T.J. (1984). Neuron-specific enolase in the diagnosis of neuroblastoma and other small, round-cell tumors in children. *Hum. Pathol.*, **15**, 575.

WEVERS, R.A., JACOBS, A.A.C. & HOMES, O.R. (1983). A bioluminescent assay for enolase (EC 4.2.1.11) activity in human serum and cerebrospinal fliud. *Clinica. Chimica. Acta.*, **135**, 159.

ZELTZER, P.M., MARANGOS, P.J., PARAMA, A.M., SATHER, H., DALTON, A., HAMMOND, D., SIEGEL, S.E. & SEEGER, R.C. (1983). Raised neuron-specific enolase in serum of children with metastatic neuroblastoma. *Lancet*, **ii**, 361.


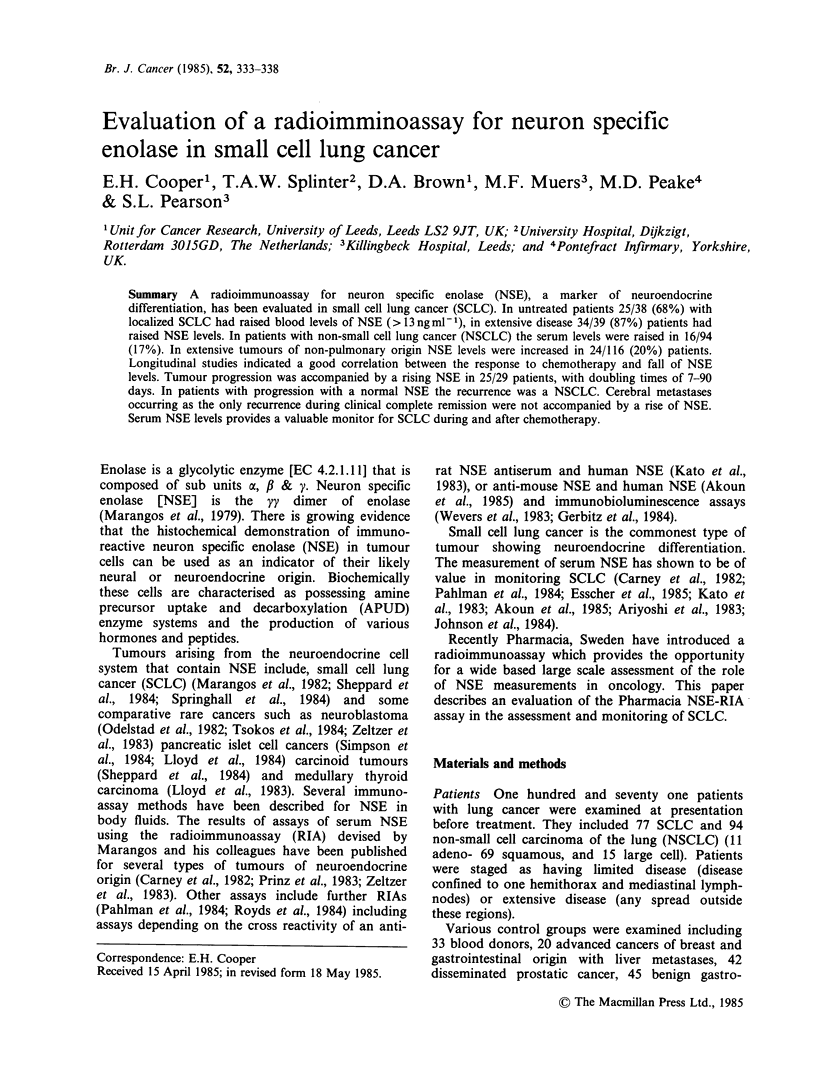

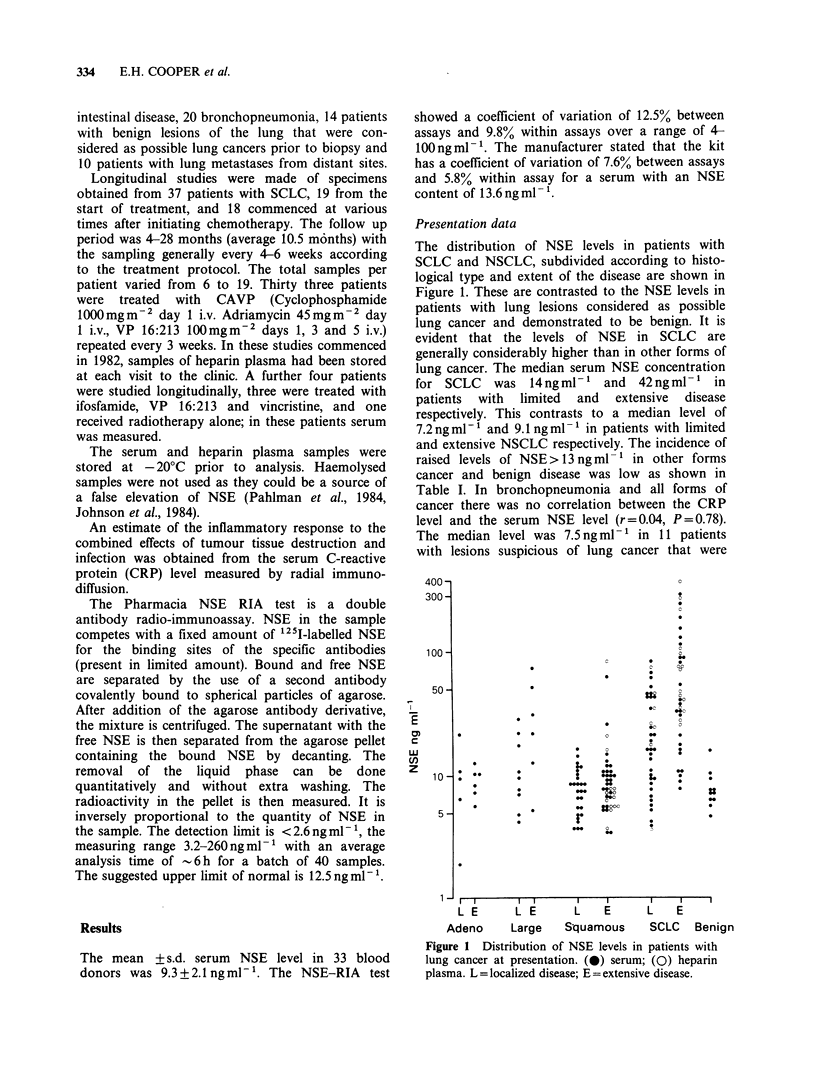

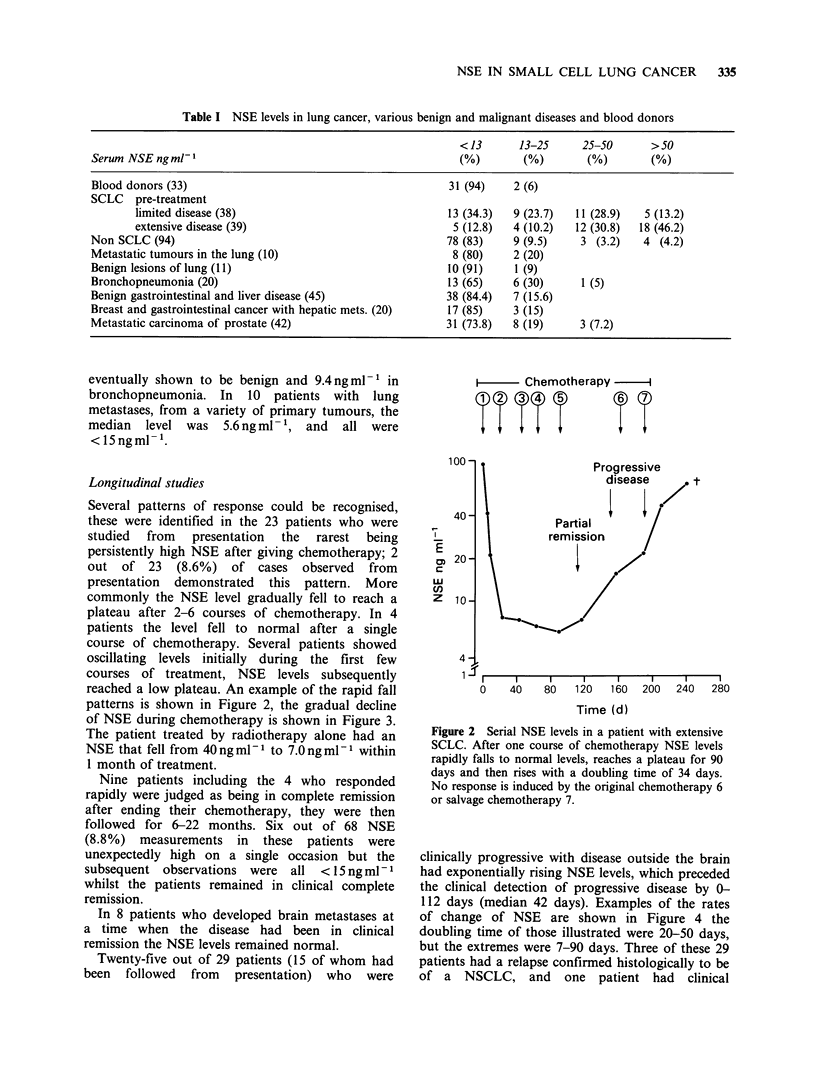

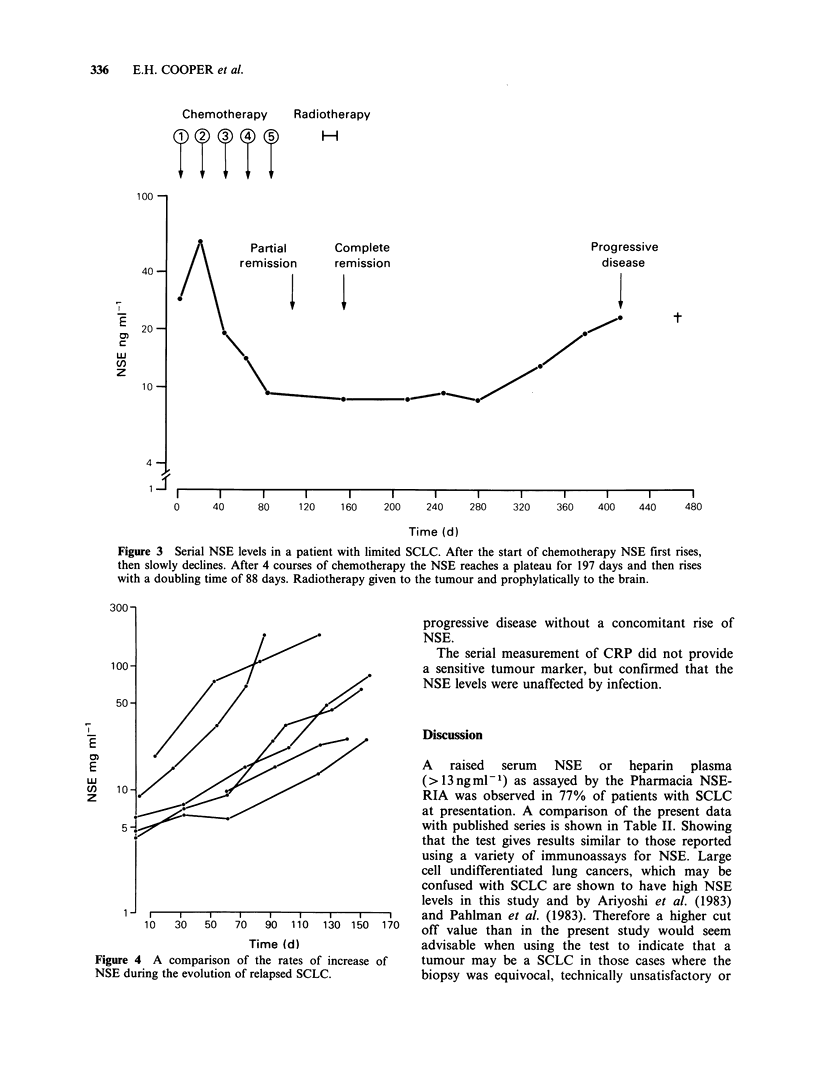

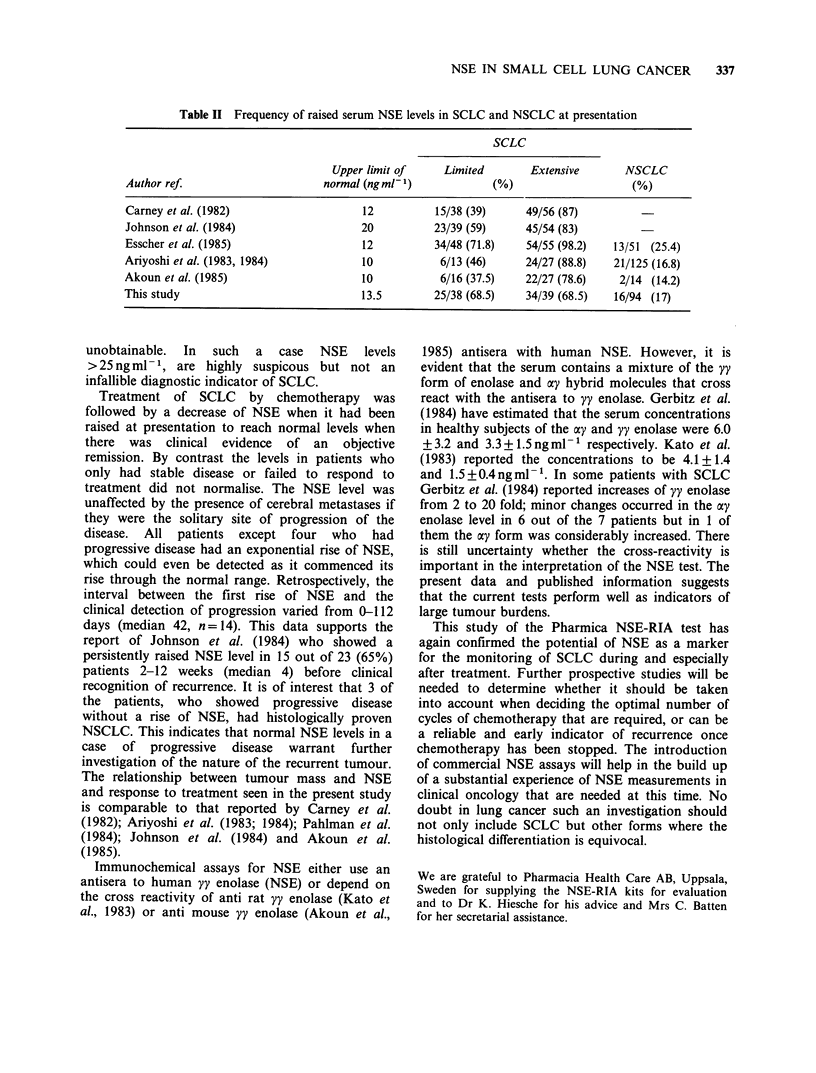

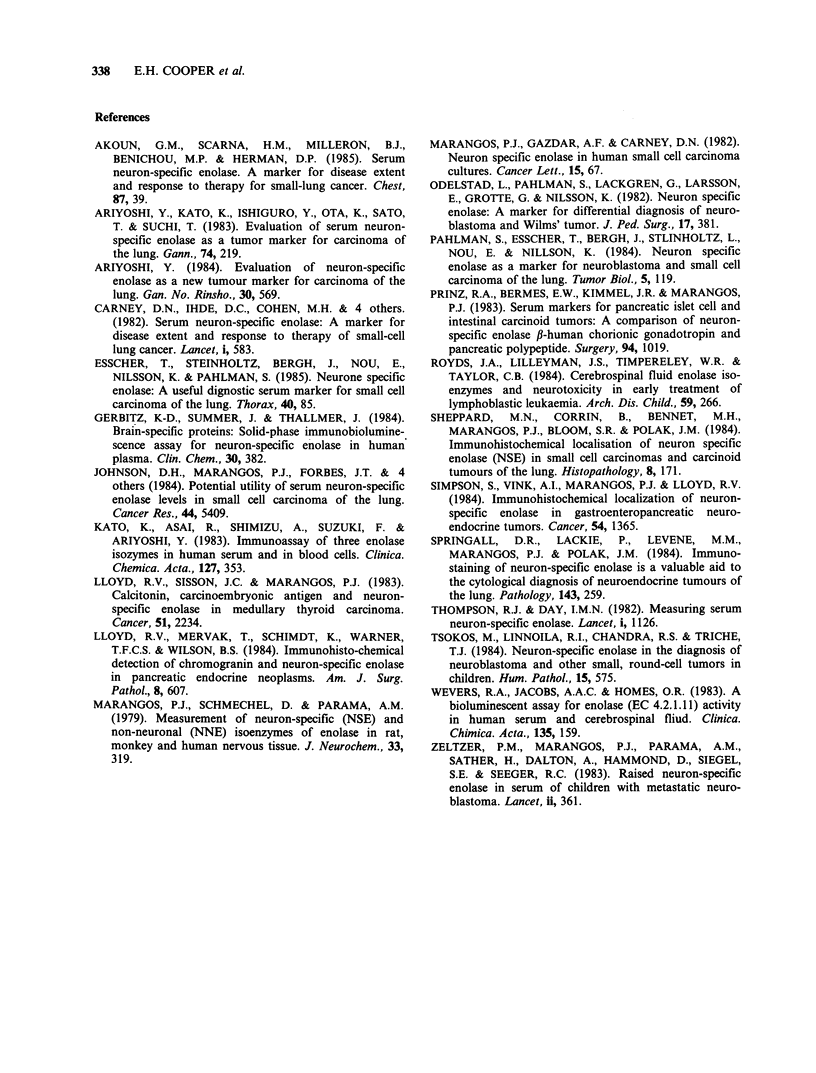


## References

[OCR_00594] Akoun G. M., Scarna H. M., Milleron B. J., Bénichou M. P., Herman D. P. (1985). Serum neuron-specific enolase. A marker for disease extent and response to therapy for small-cell lung cancer.. Chest.

[OCR_00601] Ariyoshi Y., Kato K., Ishiguro Y., Ota K., Sato T., Suchi T. (1983). Evaluation of serum neuron-specific enolase as a tumor marker for carcinoma of the lung.. Gan.

[OCR_00607] Ariyoshi Y. (1984). [Evaluation of neuron-specific enolase as a new tumor marker for carcinoma of the lung].. Gan No Rinsho.

[OCR_00612] Carney D. N., Marangos P. J., Ihde D. C., Bunn P. A., Cohen M. H., Minna J. D., Gazdar A. F. (1982). Serum neuron-specific enolase: a marker for disease extent and response to therapy of small-cell lung cancer.. Lancet.

[OCR_00618] Esscher T., Steinholtz L., Bergh J., Nöu E., Nilsson K., Påhlman S. (1985). Neurone specific enolase: a useful diagnostic serum marker for small cell carcinoma of the lung.. Thorax.

[OCR_00624] Gerbitz K. D., Summer J., Thallemer J. (1984). Brain-specific proteins: solid-phase immunobioluminescence assay for neuron-specific enolase in human plasma.. Clin Chem.

[OCR_00630] Johnson D. H., Marangos P. J., Forbes J. T., Hainsworth J. D., Van Welch R., Hande K. R., Greco F. A. (1984). Potential utility of serum neuron-specific enolase levels in small cell carcinoma of the lung.. Cancer Res.

[OCR_00636] Kato K., Asai R., Shimizu A., Suzuki F., Ariyoshi Y. (1983). Immunoassay of three enolase isozymes in human serum and in blood cells.. Clin Chim Acta.

[OCR_00648] Lloyd R. V., Mervak T., Schmidt K., Warner T. F., Wilson B. S. (1984). Immunohistochemical detection of chromogranin and neuron-specific enolase in pancreatic endocrine neoplasms.. Am J Surg Pathol.

[OCR_00642] Lloyd R. V., Sisson J. C., Marangos P. J. (1983). Calcitonin, carcinoembryonic antigen and neuron-specific enolase in medullary thyroid carcinoma.. Cancer.

[OCR_00662] Marangos P. J., Gazdar A. F., Carney D. N. (1982). Neuron specific enolase in human small cell carcinoma cultures.. Cancer Lett.

[OCR_00655] Marangos P. J., Schmechel D., Parma A. M., Clark R. L., Goodwin F. K. (1979). Measurement of neuron-specific (NSE) and non-neuronal (NNE) isoenzymes of enolase in rat, monkey and human nervous tissue.. J Neurochem.

[OCR_00667] Odelstad L., Påhlman S., Läckgren G., Larsson E., Grotte G., Nilsson K. (1982). Neuron specific enolase: a marker for differential diagnosis of neuroblastoma and Wilms' tumor.. J Pediatr Surg.

[OCR_00679] Prinz R. A., Bermes E. W., Kimmel J. R., Marangos P. J. (1983). Serum markers for pancreatic islet cell and intestinal carcinoid tumors: a comparison of neuron-specific enolase, beta-human chorionic gonadotropin and pancreatic polypeptide.. Surgery.

[OCR_00673] Påhlman S., Esscher T., Bergh J., Steinholtz L., Nöu E., Nilsson K. (1984). Neuron-specific enolase as a marker for neuroblastoma and small-cell carcinoma of the lung.. Tumour Biol.

[OCR_00686] Royds J. A., Lilleyman J. S., Timperley W. R., Taylor C. B. (1984). Cerebrospinal fluid enolase isoenzymes and neurotoxicity in early treatment of lymphoblastic leukaemia.. Arch Dis Child.

[OCR_00692] Sheppard M. N., Corrin B., Bennett M. H., Marangos P. J., Bloom S. R., Polak J. M. (1984). Immunocytochemical localization of neuron specific enolase in small cell carcinomas and carcinoid tumours of the lung.. Histopathology.

[OCR_00705] Springall D. R., Lackie P., Levene M. M., Marangos P. J., Polak J. M. (1984). Immunostaining of neuron-specific enolase is a valuable aid to the cytological diagnosis of neuroendocrine tumours of the lung.. J Pathol.

[OCR_00712] Thompson R. J., Day I. N. (1982). Measuring serum neurone-specific enolase.. Lancet.

[OCR_00716] Tsokos M., Linnoila R. I., Chandra R. S., Triche T. J. (1984). Neuron-specific enolase in the diagnosis of neuroblastoma and other small, round-cell tumors in children.. Hum Pathol.

[OCR_00722] Wevers R. A., Jacobs A. A., Hommes O. R. (1983). A bioluminescent assay for enolase (EC 4.2.1.11) activity in human serum and cerebrospinal fluid.. Clin Chim Acta.

[OCR_00728] Zeltzer P. M., Marangos P. J., Parma A. M., Sather H., Dalton A., Hammond D., Siegel S. E., Seeger R. C. (1983). Raised neuron-specific enolase in serum of children with metastatic neuroblastoma. A report from the Children's Cancer Study Group.. Lancet.

